# ﻿*Strobilanthes danxiaensis*, a new species of Acanthaceae endemic to Danxia Mountain in Guangdong Province, China

**DOI:** 10.3897/phytokeys.268.172546

**Published:** 2025-12-16

**Authors:** Bao-Huan Wu, Ji-Fang Zhang, Jian-Qiang Guo, Wei Wang, Se-Ping Dai, Guo-Feng Liu, Qiang Fan

**Affiliations:** 1 Guangzhou Institute of Forestry and Landscape Architecture, Guangzhou 510405, China Guangzhou Institute of Forestry and Landscape Architecture Guangzhou China; 2 Guangzhou Collaborative Innovation Center on Science-Tech of Ecology and Landscape, Guangzhou 510405, China Guangzhou Collaborative Innovation Center on Science-Tech of Ecology and Landscape Guangzhou China; 3 Administrative Commission of Danxiashan National Park, Shaoguan 512300, China Administrative Commission of Danxiashan National Park Shaoguan China; 4 Yunluo Botanical Garden, Guangzhou 510304, China Yunluo Botanical Garden Guangzhou China; 5 Guangdong Provincial Key Laboratory of Plant Resources, School of Life Sciences, Sun Yat-Sen University, Guangzhou 510275, China Sun Yat-Sen University Guangzhou China

**Keywords:** Biodiversity, Shaoguan, taxonomy

## Abstract

*Strobilanthes danxiaensis*, a new species endemic to the Danxia Mountain region of Guangdong, China, is described herein. Phylogenetic analysis based on nuclear ITS sequences indicates that *S. danxiaensis*, *S. japonica*, *S. tetrasperma* and *S. austrosinensis* form a well-supported clade. Morphologically, *S. danxiaensis* is most similar to *S. tetrasperma* and *S. austrosinensis*; however, it is readily distinguished from *S. tetrasperma* by its longer corolla, much broader oblong–obovate and emarginate corolla lobes, and a longer style. It further differs from *S. austrosinensis* in having larger oblong–obovate, emarginate corolla lobes, shorter filaments, and a longer style. The integration of morphological and molecular evidence supports the recognition of *S. danxiaensis* as a distinct species.

## ﻿Introduction

Acanthaceae is a large family with approximately 220 genera and over 4,000 species, widely distributed in tropical and subtropical regions (Hu and Tsui 2002, Hu et al. 2011, Deng 2019). *Strobilanthes* Blume, as the second most species-rich genus within Acanthaceae, comprises more than 400 species (Hu and Tsui 2002; Hu et al. 2011; Deng 2019; Chen et al. 2020). The taxonomic history of *Strobilanthes* is complex, challenged by the high diversity in its vegetative and reproductive traits. Historically, some taxonomists segregated it into numerous smaller genera based on morphological characters (Bremekamp 1944, Carine and Scotland 2002), but molecular phylogenetic studies have suggested that these segregate genera should be placed together under a broader concept of *Strobilanthes* (Moylan et al. 2004). This broader concept has been increasingly accepted by the scientific community (Deng et al. 2006; Wood and Scotland 2009, 2021; Hu et al. 2011; Wood 2014). *Strobilanthes* species are primarily distributed in tropical and subtropical regions of Asia, with China being one of the centers of diversity for the genus, having more than 120 species recorded, many of which are endemic (Hu et al. 2011).

Danxia Mountain in Guangdong represents a typical Danxia landform, characterized by steep cliffs, isolated peaks, deep ravines, and a striking mosaic of microhabitats ranging from exposed rocky slopes to humid valley bottoms. These geomorphological and microclimatic conditions have supported high levels of plant diversity and local endemism. In recent years, several new species have been continuously discovered and described from this region (Zhao et al. 2021; Fu et al. 2022; Xu et al. 2022; Huang et al. 2023; Wang et al. 2023). In the past two years, we discovered an additional species of *Strobilanthes* in Danxia Mountain, and subsequent study confirmed that it represents a new species, which is formally described in this paper.

## ﻿Materials and methods

### ﻿Field survey and specimen collection

Multiple field surveys were conducted in the Danxia Mountain region of Guangdong Province from 2024 to 2025. During these surveys, we recorded detailed ecological information about the new species, including its habitat and co-occurring plants. We also made on-site observations, measuring and taking photographs of the plant’s morphological features, such as leaves, inflorescence, corolla, stamens, and fruits. Type specimens were collected and deposited in the herbarium of Sun Yat-sen University (SYS) and the herbarium of South China Botanical Garden (IBSC).

### ﻿Morphological comparison and literature review

To confirm the taxonomic status of the new species, we conducted a detailed morphological comparison of the collected specimens with other *Strobilanthes* species from China and neighboring regions. Morphological data of the new species were measured by rulers and recorded based on nine individuals, and the comparison was conducted primarily using descriptions in relevant literature (Bremekamp 1944; Carine and Scotland 2002; Hu and Tsui 2002; Deng et al. 2006; Deng et al. 2009; Wood and Scotland 2009, 2021; Hu et al. 2011; Chen et al. 2019; Thomas et al. 2019; Nilanthi et al. 2023; Hu et al. 2024; Kladwong and Chantaranothai 2024) as well as online herbarium resources (e.g. the Chinese Virtual Herbarium, CVH).

### ﻿Molecular phylogenetic analysis

A total of 40 samples representing 37 species of *Strobilanthes* and one species of *Ruellia* L. were sampled for phylogenetic analysis, including the newly described species. Among these, the new species, *S. austrosinensis* Y.F.Deng & J.R.I.Wood and *S. tetrasperma* (Champ. ex Benth.) Druce were newly collected and sequenced for this study. The remaining 36 samples were represented by ITS sequences downloaded from GenBank. GenBank accession numbers for all sequences, including newly generated data, are listed in Table 1. *Ruellia brittoniana* Leonard was selected as the outgroup based on previous phylogenetic studies (Fernandes et al. 2025).

**Table 1.** GenBank accession numbers of the sequences used in this study.

Species	Accession No.	Species	Accession No.
*Strobilanthes danxiaensis*	PX368963	*S. isophylla*	AY489352
*S. austrosinensis*	PX368964	*S. japonica*	AY489356
*S. tetrasperma* 1	PX532389	*S. kunthiana*	AY489377
*S. tetrasperma* 2	PX532390	*S. lamiifolia*	AY489347
*S. alata*	AY489360	*S. lawsonii*	MT896161
*S. anamallaica*	MT876221	*S. lawsonii*	AY489375
*S. anceps*	AY489395	*S. lupulina*	AY489397
*S. andamanensis*	AY489386	*S. micrantha*	AY489388
*S. asper*	AY489399	*S. neilgherrensis*	AY489373
*S. attenuata*	AY489344	*S. pulcherrima*	AY489368
*S. barbata*	AY489382	*S. punctata*	AY489396
*S. bibracteata*	AY489359	*S. repanda*	AY489366
*S. cernua*	AY489361	*S. rubicunda*	AY489370
*S. ciliata*	AY489381	*S. steenisiana*	AY489357
*S. decurrens*	AY489390	*S. stenodon*	AY489372
*S. filiformis*	AY489353	*S. versicolor*	MT914278
*S. foliosa*	OM436003	*S. virendrakumarana*	MT896160
*S. habracanthoides*	AY489369	*S. walkeri*	AY489391
*S. imbricata*	AY489362	*S. walkeri*	MT889663
*S. involucrata*	AY489358	*Ruellia brittoniana*	EF214458

For the newly sequenced samples, fresh leaf material was silica-gel-dried in the field, and total genomic DNA was extracted using the CTAB protocol (Doyle and Doyle 1987). The extracted DNA was sent to Jierui Biotech (Guangzhou, China) for sequencing on the Illumina NovaSeq X Plus platform in PE150 mode. Raw reads were assembled into complete ribosomal DNA (rDNA) contigs using GetOrganelle v1.7.5 (Jin et al. 2020). The nuclear ribosomal internal transcribed spacer (ITS) region was extracted from the assembled rDNA contigs using BLAST+ (Camacho et al. 2009). A dataset was then compiled by combining the newly generated ITS sequences with all 36 publicly available ITS accessions in GenBank.

Sequences were aligned using MAFFT v7.453 (Nakamura et al. 2018) with the L-INS-i algorithm, and ambiguously aligned regions were excluded using Gblocks 0.91b (Talavera and Castresana 2007) with the strict-plus option. Maximum-likelihood (ML) phylogenetic inference was conducted using IQ-TREE v1.6.12 (Nguyen et al. 2015) under the best-fitting substitution model (TNe+R3), selected by ModelFinder (Kalyaanamoorthy et al. 2017) based on the Bayesian information criterion. Branch support was assessed with 10,000 ultrafast bootstrap replicates. The phylogenetic tree was visualized and annotated using the Interactive Tree of Life (iTOL) v6 (Letunic and Bork 2024).

## ﻿Results

### ﻿Morphological characteristics of the new species

*Strobilanthes danxiaensis* is a perennial herb up to 80 cm tall. It is morphologically similar to *Strobilanthes tetrasperma* and *S. austrosinensis* by having isophyllous leaves that are ovate to elliptic with an attenuate base and acute apex, pubescent corolla, calyx subequally 5-lobed to the base, 4-seeded capsule, and erect anthers. However, it differs from *S. tetrasperma* in having a longer corolla (2.8–4 cm vs. 1.5–2 cm), larger corolla lobes that are oblong–obovate, ca. 1 × 1 cm and emarginate (vs. oblong, ca. 4 × 5 mm and apex obtuse), and a longer style (3 cm vs. 1.5 cm). Compared to *S. austrosinensis*, *S. danxiaensis* has larger oblong–obovate, emarginate corolla lobes (ca. 1 × 1 cm vs. ovate-elliptic, ca. 5 mm in length), shorter filaments (shorter pair ca. 2 mm, longer pair ca. 6 mm vs. shorter pair 6 mm, longer pair 8 mm), and a longer style (3 cm vs. 1.5 cm). These combined characters clearly separate *S. danxiaensis* from both of its closest relatives. A more detailed morphological comparison of these species was summarized in Table 2.

**Table 2.** Morphological comparison of *Strobilanthes danxiaensis*, *S. austrosinensis* and *S. japonica*.

Character	*Strobilanthes danxiaensis*	*S. tetrasperma*	*S. austrosinensis*	*S. japonica*
Height	up to 80 cm	30–50 cm	to 50 cm	20–50 cm
Stem	white tomentose	retrorsely pubescent along sulci, glabrescent	bifariously pubescent with reflexed large-celled trichomes	glabrous
Leaf blade	ovate to elliptic, 6.5–10 × 3.8–5.5 cm	ovate, elliptic, or oblong, 1.5–11 × 1–4.5 cm	elliptic to suborbicular, 2–8 × 1–4.5 cm	narrowly elliptic to lanceolate, 2–5 × 0.5–1.8 cm
Leaf adaxial surface	sparsely puberulous	glabrous	pilose and hirsute	glabrous
Leaf abaxial surface	tomentose along veins	glabrous	pilose along veins	glabrous
Petiole	0.3–1.1 cm, tomentose	0.5–2.5 cm, sulcate, glabrous	0–2 cm, bifariously hirsute	2–5 cm, pubescent
Bracts	leaflike, obovate to obovate-spatulate, 1–2 × 0.5–1 cm	leaflike, obovate to spatulate, 1–1.5 × 0.4–0.5 cm	leaflike, broadly obovate-spatulate, 1.5–1.8 × 0.5–0.8 cm	imbricate, oblanceolate to oblong-lanceolate, 7–10 × 2–3 mm
Bracteoles	linear to oblanceolate, 0.8–0.9 × 0.1–0.2 cm	linear, 5–6 × ca. 1 mm	spatulate, 8–11 × 1–2.5 mm	linear, ca. 5 mm
Calyx	0.8–1.1 cm long, densely pubescent on both surfaces	0.5–0.7 cm long, glabrous except for large-celled trichomes on margin, densely covered with cystoliths	1–1.2 cm long, outside pilose, inside subglabrous, margin ciliate	0.6–0.7 cm long, margin ciliate
Corolla	pale purple to whitish, 2.8–4 cm	purple to purplish blue, 1.5–2 cm	bluish purple, 2.2–2.8 cm	purplish white or white, ca. 1.5 cm
Corolla lobes	oblong to obovate, ca. 10 × 10 mm, emarginate at apex	oblong, ca. 4 × 5 mm, apex obtuse	ovate-elliptic, ca. 5 mm long	oblong-elliptic, 5–6 mm long
Filaments	shorter pair ca. 2 mm, longer pair ca. 6 mm	shorter pair ca. 2 mm, longer pair ca. 5 mm	shorter pair ca. 6 mm, longer pair ca. 8 mm	shorter pair ca. 8 mm, longer pair ca. 12 mm
Ovary	cylindrical, upper 2/3 pubescent	ovoid, ca. 3 mm, hispid at tip	ovoid, glabrous except comose tip	glabrous
Style	ca. 3 cm	ca. 1.5 cm	ca. 1.5 cm	ca. 1.2 cm
Phenology	flowering November–January, fruiting march	flowering July–December	flowering July–September, fruiting October–November	flowering August–September, fruiting October–November

### ﻿Phylogenetic analysis

**Figure 1.**
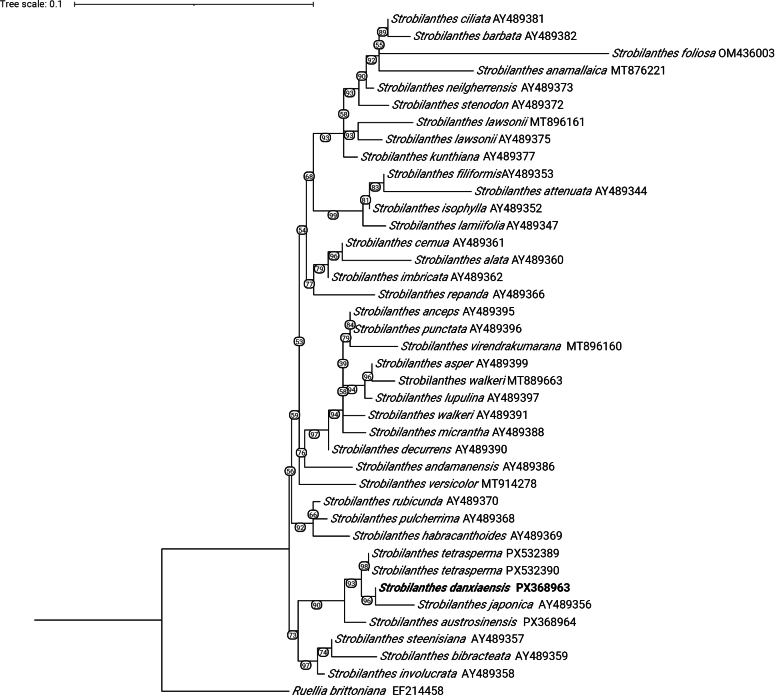
Maximum likelihood tree for the *Strobilanthes* based on the ITS dataset. Bootstrap values are labeled under the branches.

After aligning the ITS sequences of 40 samples using MAFFT and extracting conserved regions with Gblocks, the final matrix contained 345 characters, of which 105 were variable and 45 were parsimony-informative. The phylogenetic analysis (Fig. 1) shows that *S. danxiaensis* forms a strongly supported clade with *S. japonica* (BS = 96%). This clade is further grouped with two samples of *S. tetrasperma* to form another well-supported clade (BS = 93%). *S. austrosinensis* forms the sister group to the clade comprising *S. danxiaensis*, *S. japonica*, and *S. tetrasperma*, with strong support (BS = 90%).

### ﻿Taxonomic treatment

#### ﻿ Strobilanthes danxiaensis

Taxon classificationPlantaeLamialesAcanthaceae

B.H.Wu, J.F.Zhang & J.Q.Guo sp. nov.

2D01A434-8C38-5096-9ECB-C9E51098FFBB

urn:lsid:ipni.org:names:77373766-1

Fig. 2

##### Type.

**China** • **Guangdong Province**: Shaoguan City, Renhua County, Danxia Mountain area, 250 m a.s.l., in moist valleys, 4 December 2024, *Jian-Qiang Guo and Bao-Huan Wu Lg2024179* (holotype: SYS!; isotype: SYS!; IBSC!).

##### Diagnosis.

Morphologically, *Strobilanthes danxiaensis* is most similar to *S. tetrasperma*, but differs in its longer corolla (2.8–4 cm vs. 1.5–2 cm), with larger, emarginate oblong–obovate corolla lobes (ca. 1 × 1 cm vs. oblong lobes ca. 4 × 5 mm with obtuse apices), and a much longer style (3 cm vs. 1.5 cm).

**Figure 2.**
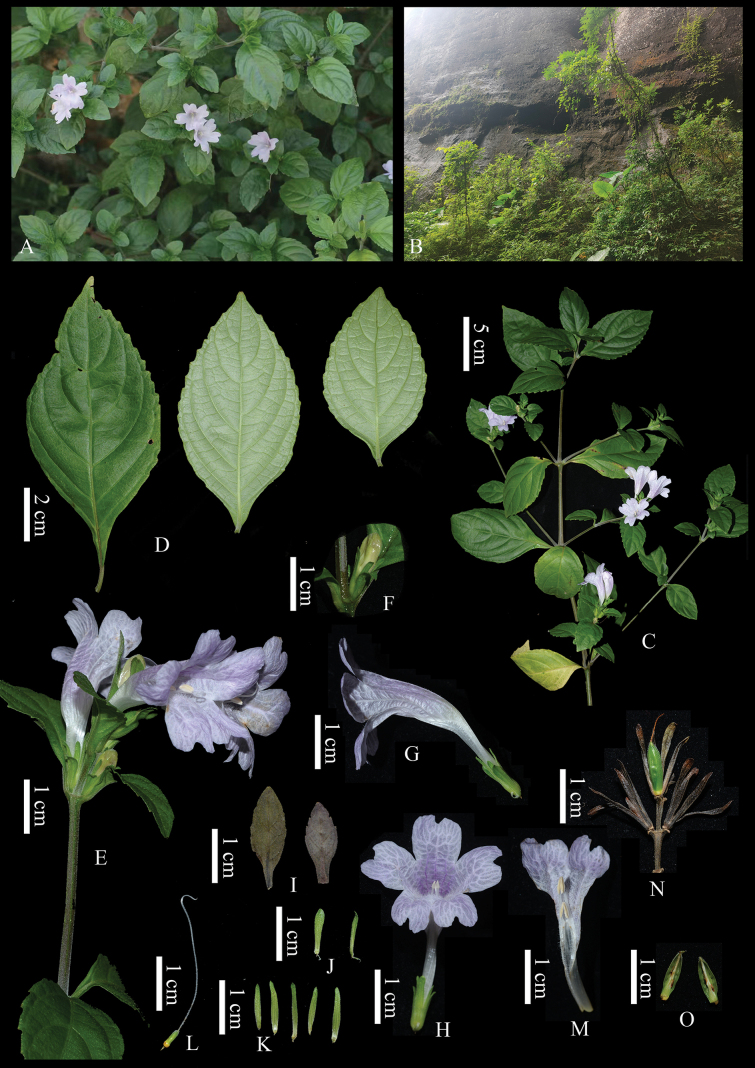
*Strobilanthes danxiaensis*. **A.** Wild individuals; **B.** Habitat; **C.** Flowering branch; **D.** Leaves; **E.** Inflorescence; **F.** Bud; **G.** Side view of flower; **H.** Front view of flower; **I.** Bracts; **J.** Bracteoles; **K.** Calyxs; **L.** Gynoecium; **M.** Dissected corolla showing stamens; **N.** Infructescence; **O.** Dissected capsule. Photographed by Jian-Qiang Guo and Bao-Huan Wu.

##### Description.

Perennial herb, up to 80 cm tall. Stems slightly prostrate at base, becoming erect or ascending upwards, quadrangular, white tomentose, gradually glabrescent below. Leaves opposite; petiole 0.3–1.1 cm long, white tomentose; blade ovate to elliptic, 6.5–10 × 3.8–5.5 cm, adaxially green and sparsely puberulous with short white hairs, cystoliths absent or sometimes sparsely present, abaxially pale green, tomentose along veins and conspicuously glandular-cystic; lateral veins 4–5(–6) pairs, prominently raised on both surfaces; base attenuate, apex acute; margin crenate-dentate with obtuse teeth. Inflorescences short terminal spikes, 1–4 cm long, often elongating at maturity, bearing 2–4 flowers; rachis tomentose. Bracts leaf-like, obovate to obovate-spatulate, 1–2 × 0.5–1 cm, puberulous on both surfaces, margin shallowly serrate, apex obtuse, base attenuate. Bracteoles linear to oblanceolate, 0.8–0.9 × 0.1–0.2 cm, midrib conspicuous, entire, densely white-hirsute on both surfaces. Calyx 5-lobed almost to base; lobes linear, 0.8–1.1 cm long, midrib prominent, densely pubescent on both surfaces, apex obtuse. Corolla pale purple to whitish, funnel-shaped, 2.8–4 cm long, slightly inflated and curved, externally puberulous, internally pubescent in throat and downward; tube basally cylindrical, ca. 2 mm wide, 1.1–1.3 cm long, gradually widened towards mouth; orifice 1.6–3 cm wide, 2–2.8 cm high; lobes oblong to obovate, subequal, ca. 1 × 1 cm, emarginate at apex. Stamens 4, included; filaments sparsely pubescent; shorter pair ca. 2 mm long, longer pair ca. 6 mm long. Anthers narrowly ellipsoid, ca. 3 mm long. Ovary cylindrical, ca. 3 mm long, upper 2/3 densely pubescent; style ca. 3 cm long, sparsely pubescent. Capsule narrowly ellipsoidal, ca. 1 cm long, ca. 2.5 mm in diam., upper 2/3 pubescent, 4-seeded.

##### Phenology.

Flowering from November to January; fruiting in March.

##### Etymology.

The specific epithet “danxiaensis” refers to the type locality, Danxia Mountain in Guangdong Province, China, to commemorate this unique landform and the rich plant diversity it harbors.

##### Distribution and habitat.

*Strobilanthes danxiaensis* is known only from Danxia Mountain, Guangdong Province, China. Only three populations have been discovered, with a total of fewer than 100 individuals. They grow in moist valleys at elevations of 350–400 m. Associated species include *Maesa perlarius* (Lour.) Merr., *Alocasia odora* (Roxb.) K. Koch, *S. dimorphotricha* Hance, *Oplismenus compositus* (L.) P. Beauv., *Tectaria devexa* Copel., and *Lobelia nummularia* Lam.

##### Conservation status.

*Strobilanthes danxiaensis* is currently known from only three small populations in moist valleys on Danxia Mountain. However, the species occurs in a rugged and heterogeneous landscape where botanical surveys remain incomplete, and additional suitable habitats in the region have not yet been systematically explored. There is insufficient evidence to formerly assess the species using IUCN guidelines and we consider this species to be Data Deficient (DD).

## ﻿Discussion

The taxonomic history of Strobilanthes has long been complex. To date, molecular systematics studies of *Strobilanthes* remain limited in both scope and taxon sampling. In this study, we used 36 ITS sequences from public databases, combined with four newly sequenced samples, to form an alignment matrix comprising 37 species and reconstructed a maximum likelihood tree. Our molecular analysis yielded unresolved relationships within *Strobilanthes*, with most clades receiving low support and interclade relationships remaining uncertain.

*Strobilanthes danxiaensis*, characterized by ovate to elliptic leaves with an attenuate base and acute apex, pubescent corollas, calyx 5-lobed to the base, 4-seeded capsules, and erect anthers, is morphologically similar to *S. tetrasperma* and *S. austrosinensis*. Because of these morphological similarities, both species were included in the phylogenetic analysis, to clarify their relationships. The molecular results support a close relationship among these species, but indicate that *S. japonica* is actually the closest relative of *S. danxiaensis*, with the two forming a strongly supported clade. This clade is subsequently united with *S. tetrasperma* and *S. austrosinensis*. Although these findings do not yet provide a definitive framework for infrageneric classification within *Strobilanthes*, they demonstrate a clear phylogenetic affinity among these four species.

Compared with its closest relative, *S. japonica*, *S. danxiaensis* has markedly larger leaves (6.5–10 × 3.8–5.5 cm vs. 2–5 × 0.5–1.8 cm), shorter petioles (0.3–1.1 cm vs. 2–5 cm), a longer corolla (2.8–4 cm vs. ca. 1.5 cm), much shorter filaments (shorter pair ca. 2 mm and longer pair ca. 6 mm vs. shorter pair ca. 8 mm and longer pair ca. 12 mm), and a longer style (ca. 3 cm vs. ca. 1.2 cm), all of which clearly differentiate the two species. Concurrently, detailed morphological comparisons corroborate that *S. danxiaensis* is readily distinguishable from *S. japonica*, *S. tetrasperma*, and *S. austrosinensis*, thereby supporting its recognition as a distinct species.

## Supplementary Material

XML Treatment for Strobilanthes danxiaensis

## ﻿Additional information

### Use of AI

No use of AI was reported.

### Author ORCIDs

Bao-Huan Wu https://orcid.org/0000-0002-0498-0603

Jian-Qiang Guo https://orcid.org/0009-0004-2304-0360

Qiang Fan https://orcid.org/0000-0003-4254-6936

### Data availability

The original contributions presented in this study are included in the article; further inquiries can be directed to the corresponding authors.

## References

[B1] Bremekamp CEB (1944) Materials for a monograph of the Strobilanthinae. Verhandelingen der Koninklijke Nederlandse Akademie van Wetenschappen. Afdeling Natuurkunde 41: 1–306.

[B2] Camacho C Coulouris G Avagyan V Ma N Papadopoulos J Bealer K Madden TL (2009) BLAST+: Architecture and applications. BMC Bioinformatics 10: 421. 10.1186/1471-2105-10-421 PMC2803857 20003500

[B3] Carine MA Scotland RW (2002) Classification of Strobilanthinae (Acanthaceae): Trying to classify the unclassifiable? Taxon 51(2): 259–279. 10.2307/1554897

[B4] Chen FL Deng YF Xiong ZB Ran JC (2019) *Strobilanthes hongii*, a new species of Acanthaceae from Guizhou, China. Phytotaxa 388(1): 135–144. 10.11646/phytotaxa.388.1.7

[B5] Chen JT Huang XH Lv ZY Kuang TH Luo J Deng YF Deng T (2020) *Strobilanthes sunhangii* (Acanthaceae), a new species from Tibet, China. PhytoKeys 166: 117–127. 10.3897/phytokeys.166.58831 33239959 PMC7679347

[B6] Deng YF (2019) Transfer of the Philippine species of *Hemigraphis* Nees to *Strobilanthes* Blume (Acanthaceae). Phytotaxa 404(5): 1–3. 10.11646/phytotaxa.404.5.3

[B7] Deng YF Wood JRI Scotland RW (2006) New and reassessed species of *Strobilanthes* (Acanthaceae) in the flora of China. Botanical Journal of the Linnean Society 150(4): 369–390. 10.1111/j.1095-8339.2006.00473.x

[B8] Deng YF Xia NH Hao ZP Sun Y (2009) Acanthaceae. In: Wu DL (Ed.) Flora of Guangdong, vol.9. Guangdong Science & Technology Press, Guangzhou, 95–154.

[B9] Doyle JJ Doyle JL (1987) A rapid DNA isolation procedure for small quantities of fresh leaf tissue. Phytochemical Bulletin 19: 11–15.

[B10] Fernandes MC Surveswaran S Sellappan K (2025) Molecular phylogeny of *Strobilanthes* (Acanthaceae) from the northern Western Ghats of India: Phylogenetic relationships and implications for systematics and biogeography. Nordic Journal of Botany 8: e04678. 10.1002/njb.04678

[B11] Fu LF Xiong C Monro AK Fan Q Chen ZX Wen F Xin ZB Wei YG Liao WB (2022) *Pilea danxiaensis* (Urticaceae), a new species in the Danxia landform from Guangdong, China including a description of the entire chloroplast genome. PhytoKeys 204: 109–119. 10.3897/phytokeys.204.86857 36760615 PMC9848946

[B12] Hu CC Tsui HP (2002) Acanthaceae. In: Hu CC (Ed.) Flora Reipublicae Popularis Sinicae, vol.70. Science Press, Beijing, 1–309. [In Chinese]

[B13] Hu JQ Deng YF Wood JRI (2011) Acanthaceae. In: Wu ZY Raven PH Hong DY (Eds) Flora of China, vol.19. Science Press & Missouri Botanical Garden Press, Beijing & St. Louis, 369–477.

[B14] Hu Y Zeng NG Zeng XH Zeng YB Peng YS Xiao DC Xu GL (2024) A new plant variety from Jiangxi Province, China: Strobilanthes dalzielii var. longnanensis (Acanthaceae). Journal of Hangzhou Normal University 23: 277–280. [Natural Science Edition]

[B15] Huang YS Meng KK Sun YY Chen ZX Fan Q (2023) A new species of *Sedum* (Crassulaceae) from Mount Danxia in Guangdong, China. PhytoKeys 221: 117–129. 10.3897/phytokeys.221.97495 37250354 PMC10209613

[B16] Jin JJ Yu WB Yang JB Song Y dePamphilis CW Yi TS Li DZ (2020) GetOrganelle: A fast and versatile toolkit for accurate de novo assembly of organelle genomes. Genome Biology 21: 241. 10.1186/s13059-020-02154-5 PMC7488116 32912315

[B17] Kalyaanamoorthy S Minh BQ Wong TKF von Haeseler A Jermiin LS (2017) ModelFinder: Fast model selection for accurate phylogenetic estimates. Nature Methods 14: 587–589. 10.1038/nmeth.4285 28481363 PMC5453245

[B18] Kladwong P Chantaranothai P (2024) Notes on *Strobilanthes* (Acanthaceae) with capitate inflorescences in Thailand. PhytoKeys 244: 89–126. 10.3897/phytokeys.244.124260 39022625 PMC11252561

[B19] Letunic I Bork P (2024) Interactive Tree of Life (iTOL) v6: Recent updates to the phylogenetic tree display and annotation tool. Nucleic Acids Research 52: W78–W82. 10.1093/nar/gkae268 PMC11223838 38613393

[B20] Moylan EC Bennett JR Carine MA Olmstead RG Scotland RW (2004) Phylogenetic relationships among *Strobilanthes* s.l. (Acanthaceae): Evidence from ITS nrDNA, trnL-F cpDNA and morphology. American Journal of Botany 91(5): 724–735. 10.3732/ajb.91.5.724 21653427

[B21] Nakamura T Yamada KD Tomii K Katoh K (2018) Parallelization of MAFFT for large-scale multiple sequence alignments. Bioinformatics (Oxford, England) 34(14): 2490–2492. 10.1093/bioinformatics/bty121 29506019 PMC6041967

[B22] Nguyen LT Schmidt HA von Haeseler A Minh BQ (2015) IQ-TREE: A fast and effective stochastic algorithm for estimating maximum-likelihood phylogenies. Molecular Biology and Evolution 32(1): 268–274. 10.1093/molbev/msu300 25371430 PMC4271533

[B23] Nilanthi RMR Gopallawa B Jayawardana N Jayasinghe HD (2023) *Strobilanthes sripadensis*, a new species of Acanthaceae from Sri Lanka. Phytotaxa 592(2): 127–134. 10.11646/phytotaxa.592.2.6

[B24] Talavera G Castresana J (2007) Improvement of phylogenies after removing divergent and ambiguously aligned blocks from protein sequence alignments. Systematic Biology 56(4): 564–577. 10.1080/10635150701472164 17654362

[B25] Thomas J Britto SJ Daniel TF (2019) *Strobilanthes tirunelveliensis* (Acanthaceae), a new species from southern India. Phytotaxa 407(1): 97–104. 10.11646/phytotaxa.407.1.9

[B26] Wang LY Zhao WY Chen ZX Huang WC Ding MY Luo JC Liao WB Guo W Fan Q (2023) *Commelina danxiaensis* (Commelinaceae), a new species from Guangdong, China. PhytoKeys 218: 117–126. 10.3897/phytokeys.218.91199 36762279 PMC9860505

[B27] Wood JRI (2014) New names and combinations in Indian Acanthaceae. Novon 23(3): 385–395. 10.3417/2013046

[B28] Wood JRI Scotland RW (2009) New and little-known species of *Strobilanthes* (Acanthaceae) from India and South East Asia. Kew Bulletin 64(1): 3–47. 10.1007/s12225-009-9098-2

[B29] Wood JRI Scotland RW (2021) A *Strobilanthes* (Acanthaceae) miscellany. Kew Bulletin 76: 827–840. 10.1007/s12225-021-09990-z

[B30] Xu KW Lin CX Guo JQ Zhou XX Liao WB Mao LF (2022) *Asplenium danxiaense* sp. nov. (Aspleniaceae, Aspleniineae), a new tetraploid fern species from Guangdong, China, based on morphological and molecular data. European Journal of Taxonomy 798: 162–173. 10.5852/ejt.2022.798.1679

[B31] Zhao WY Jiang KW Chen ZX Tian B Fan Q (2021) *Lespedeza danxiaensis* (Fabaceae), a new species from Guangdong, China, based on molecular and morphological data. PhytoKeys 185: 43–53. 10.3897/phytokeys.185.72788 34819780 PMC8608780

